# Above and belowground community strategies respond to different global change drivers

**DOI:** 10.1038/s41598-019-39033-4

**Published:** 2019-02-22

**Authors:** Karen L. Adair, Stinus Lindgreen, Anthony M. Poole, Laura M. Young, Maud Bernard-Verdier, David A. Wardle, Jason M. Tylianakis

**Affiliations:** 1 School of Biological Sciences, University of Canterbury, Private Bag 4800, Christchurch, 8140 New Zealand; 2 Present Address: Department of Entomology, Comstock Hall, Cornell University, Ithaca, 14853 NY USA; 3 Present Address: H. Lundbeck A/S, Ottiliavej 9, 2500 Valby, Denmark; 4 Present Address: School of Biological Sciences, University of Auckland, Private Bag 92019, Auckland, 1142 New Zealand; 5 Bio-Protection Research Centre, Lincoln University, PO Box 85084, Lincoln, 7647 Canterbury New Zealand; 6 Present Address: Freie Universität Berlin, Institut für Biologie, Königin-Luise-Str. 1-3, 14195 Berlin-Dahlem, Germany; 7 Department of Forest Ecology and Management, Swedish University of Agricultural Sciences, SE901-83 Umea, Sweden; 8 Asian School of the Environment, Nanyang Technological University, 50 Nanyang Avenue, Singapore, 639798 Singapore; 9 Department of Life Sciences, Imperial College London, Silwood Park Campus, Buckhurst Road, Ascot, Berkshire SL5 7PY United Kingdom

## Abstract

Environmental changes alter the diversity and structure of communities. By shifting the range of species traits that will be successful under new conditions, environmental drivers can also dramatically impact ecosystem functioning and resilience. Above and belowground communities jointly regulate whole-ecosystem processes and responses to change, yet they are frequently studied separately. To determine whether these communities respond similarly to environmental changes, we measured taxonomic and trait-based responses of plant and soil microbial communities to four years of experimental warming and nitrogen deposition in a temperate grassland. Plant diversity responded strongly to N addition, whereas soil microbial communities responded primarily to warming, likely via an associated decrease in soil moisture. These above and belowground changes were associated with selection for more resource-conservative plant and microbe growth strategies, which reduced community functional diversity. Functional characteristics of plant and soil microbial communities were weakly correlated (*P* = 0.07) under control conditions, but not when above or belowground communities were altered by either global change driver. These results highlight the potential for global change drivers operating simultaneously to have asynchronous impacts on above and belowground components of ecosystems. Assessment of a single ecosystem component may therefore greatly underestimate the whole-system impact of global environmental changes.

## Introduction

Ecosystems are responding to multiple simultaneous environmental changes. These changes alter the diversity and composition of communities^1,2^ and shift the range of species traits that will be successful, thereby dramatically impacting ecosystem functioning and resilience^3,4^. Above and belowground communities jointly regulate whole-ecosystem processes and responses to environmental change^5,6^. However, most studies quantify responses of a single ecosystem component, and it is unclear whether results of such studies can be extrapolated to the rest of the ecosystem. There is evidence that plant and soil microbial diversity are frequently correlated at different spatial scales^7^, and that microbes can mediate plant responses to drivers such as nutrient enrichment^8^. However, differential sensitivity of plant and microbial communities^9^ suggests that these communities may have distinct responses to environmental factors.

Multiple processes determine plant and soil microbial community responses to global change. Global change drivers shift the range of species traits that will be successful and thus can directly influence the diversity and composition of both plant and soil microbial communities. However, shifts in the traits of either community can alter the interactions between plants and soil microbes, and changes in these linkages can further influence the responses of both communities. For example, nitrogen addition typically results in decreased diversity of plant communities^2,10,11^ and, after initial increases in productivity, this loss of species can be associated with decreased productivity in the longer term^12^. Warming tends to be associated with increases in aboveground biomass^13,14^ and shifts in plant community composition^15^. Such changes in the plant community will alter the quantity and quality of inputs to soil microbial communities and impact upon the decomposition and nitrogen cycling processes that they regulate^16–21^. However, global change drivers can also have direct impacts on soil communities. Both nitrogen addition and warming have been shown to decrease soil microbial biomass^22–25^ and to shift the composition and functional capacity of soil bacterial communities^26–32^, thus altering rates of soil nutrient cycling which in turn regulate nutrient availability to plants. To more completely understand terrestrial ecosystem responses to global change, both plant and soil communities must be considered simultaneously.

For both plants and microbes, multiple species may perform the same ecosystem role or respond similarly to environmental drivers, meaning that shifts in taxonomic diversity may not capture functionally important changes. Consequently, species traits have been embraced as a means to measure functionally important changes within plant communities^33^, and calls have been made for the use of similar approaches for microbes^34,35^. Such parallel approaches could be crucial, particularly if environmental drivers select for different traits in plant versus soil microbial communities. Finally, the simultaneous effects of multiple environmental changes may be non-additive^2,36,37^, because different drivers can select for either similar or incompatible sets of species traits. Therefore, establishing whether suites of environmental changes drive parallel shifts above and belowground is pivotal to predicting how ecosystems will respond to future global change.

Here we quantify how the composition (taxonomy) and functional characteristics (traits) of above and belowground communities respond to the interactive impact of two major drivers of global environmental change: increasing temperature and nitrogen (N) deposition. We measured the responses of colonizing plant and soil microbial communities in an experimentally-planted subalpine tussock grassland in the South Island of New Zealand after four years of experimental treatments applied in a factorial design: (i) soil warming by a mean of 3 °C above ambient, which led to soil moisture levels nearly 20% lower than in control plots (Table S1), and (ii) N addition (ammonium nitrate equivalent to 50 kg N ha^−1^ yr^−1^)^38,39^. Details of taxonomy and trait measurements for the plant and soil microbial communities are provided in Table S2.

## Results and Discussion

**Figure 1:**
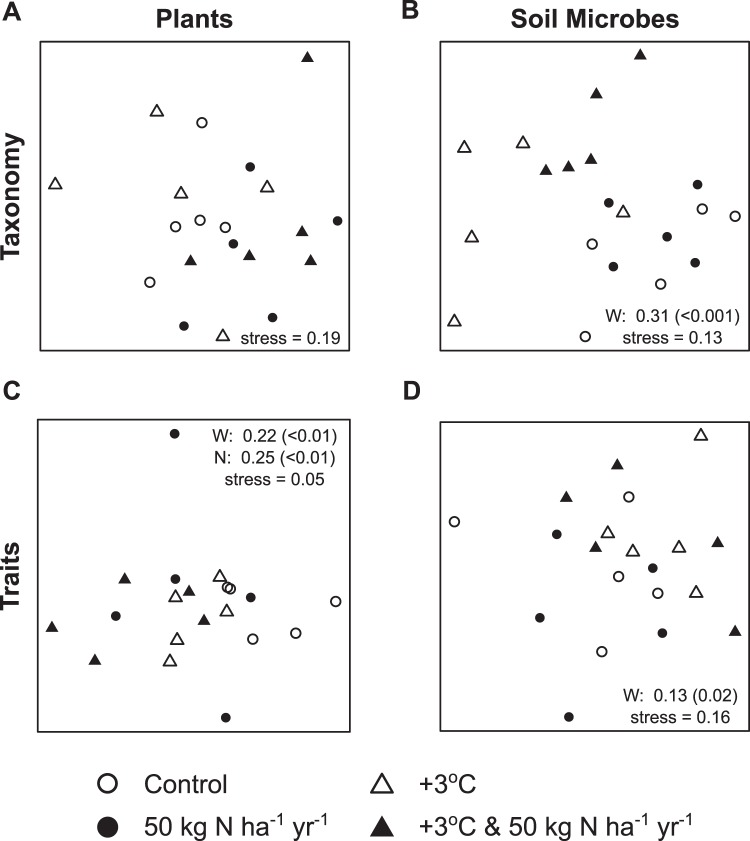
Plant and soil microbial community composition responses to global change drivers. Non-metric multidimensional scaling (NMDS) ordinations of experimental plots based on Bray-Curtis dissimilarities calculated from: (**A**) percent cover of colonizing plant species, (**B**) relative abundance of bacterial phyla, (**C**) community weighted mean trait values of the colonizing plant species, and (**D**) relative abundance of the broadest level of subsystems of microbial protein-coding genes. Significant results (*p* < 0.05) from permutational multivariate analysis of variance (permanova) tests for the same datasets are listed. Partial *R*^2^ values are given with *p*-values listed in parentheses for those results with *p* < 0.05 (W: warming, N: nitrogen addition, N × W: nitrogen and warming). Complete permanova results are given in Table S3. See Supplementary Information for equivalent ordinations (Fig. S1) and permanova results (Table S7) for the relative abundance of bacterial genera and the most specific level of bacterial protein-coding genes.

**Figure 2:**
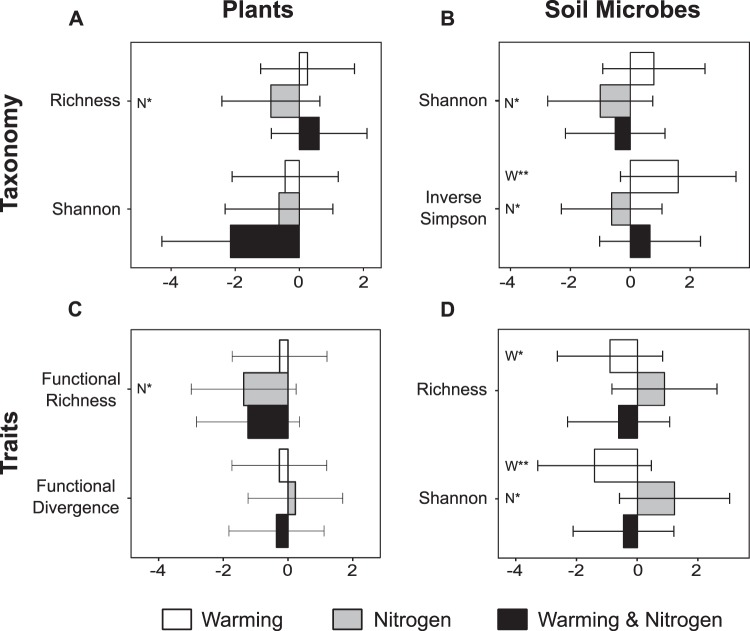
Plant and soil microbial diversity responses to global change drivers based on taxonomy and traits. Effect sizes (Cohen’s d) and 95% confidence intervals for each treatment combination relative to control plots for: (**A**) richness and Shannon diversity index calculated from percent cover of colonizing plant species, (**B**) Shannon and inverse Simpson diversity indices calculated from the relative abundance of bacterial phyla, (**C**) functional richness and functional divergence calculated from trait values of the colonizing plant species, and (**D**) richness and Shannon diversity index calculated from relative abundance of functional roles (most specific categories of protein-coding genes). Significant treatment effects from linear models are indicated by N for nitrogen addition, W for soil warming, and W × N for the interactive effect. * indicates *p* < 0.05, ** indicates, *p* < 0.01, and *** indicates *p* < 0.001. Details for other diversity metrics and statistical analyses are provided in Table S4.

We first assessed whether the diversity and composition of above and belowground communities responded similarly to warming and N addition. For plants, we assessed separately the responses of the planted and the colonizing community, as the latter underwent stronger environmental filtering than the former, and the colonizing community indicates the trajectory of the community. Neither treatment significantly altered the composition of the colonizing plant community based on percent cover (Fig. 1A, Table S3), but nitrogen addition did reduce the number of colonizing plant species (Fig. 2A, Table S4), in line with many previous studies^2,10,11^. Because richness of the planted community was constant across treatments, this reduction in species richness applied to both the colonizing and total community. Nitrogen addition promoted growth of the planted tussock *Chionochloa rigida*, and reduced abundance of colonizing *Trifolium repens*, the dominant N-fixing species (Table S5). The total cover of all plants with N-fixing associations was also significantly reduced under N addition (Table S4), and since the planted species were not N-fixers, this reduction applied to both the colonizing component and the entire community. There were no significant treatment impacts on the cover of any other abundant plant functional group (Table S5). In contrast to the pronounced effects of N addition, soil warming did not significantly impact diversity of the colonizing plant community (Fig. 2A, Table S4). The only plant species to show individual responses to soil warming were the planted tussock species *C. rigida*, which had increased cover, and *C. flavecens*, which had an interactive response to the two drivers (Table S5). Previous research has also found that *C. rigida* and *C. flavescens* have differing responses to nutrient availability^40^. Specifically, in line with our study they found that *C. rigida* biomass increased significantly with N addition and *C. flavescens* only responded to N addition when phosphate was also supplemented^40^. The diversity of the colonizing plants was not correlated with cover of the planted tussock species (Table S6).

While we observed strong plant community responses to N addition, soil bacterial communities responded primarily to warming. Soil warming, but not N addition, significantly influenced soil bacterial community composition (Fig. 1B, Table S3). This warming effect remained consistent regardless of the level of taxonomic resolution used (Fig. S1A, Table S7). Specifically, the relative abundances of Actinobacteria and Firmicutes increased with soil warming, while Bacteroidetes and Proteobacteria declined (Figs S2,S3), consistent with shifts in soil microbial communities observed after 12 years warming in a temperate hardwood forest^24^. These changes suggest a shift toward a community better able to tolerate decreasing soil moisture (80% of ambient in warmed plots), consistent with prior work pointing to microbial responses to precipitation manipulations in the field^9,41,42^ and variation in the soil moisture niches of these phyla^34^. Conversely, N addition did not significantly alter the composition of the soil bacterial communities (Figs 1B and S1A, Tables S3 and S7). Changes in the diversity of the soil bacterial community were very small and only detected for bacterial phyla and not genera (Fig. 2B, Table S4). Addition of N and soil warming had no significant interactive effects on the taxonomic composition or diversity of either plant or soil bacterial communities, suggesting that the effects of the two drivers were additive. This contrasts with earlier work from this experiment showing no main effect of warming or nitrogen on microbial biomass, but an interactive effect of the two drivers which reduced microbial biomass^39^. Combined, these results suggest that the number and relative abundance of taxa may have changed despite no overall change in total biomass, and vice versa.

We next assessed how functional characteristics of the plant and soil microbial communities, measured respectively as plant functional traits^43^ and as the relative abundance of functional categories of microbial protein-coding genes^35^, responded to N addition and soil warming. Nitrogen addition and soil warming independently influenced the overall ecological strategy of the plant communities that colonized the plots, as revealed by multivariate analyses of standardized community weighted mean trait values (Fig. 1C, Table S3). Plant communities colonizing N addition plots had significantly lower functional richness (Fig. 2C, Table S4), observed as communities that occupy less functional trait space^44^. These changes in functional diversity of the colonizing community were not correlated with changing cover of the planted tussocks (Table S6). The decrease in functional richness suggests that N addition excludes plants with extreme trait values resulting in less functionally diverse plant communities, which are likely to show decreased resilience to disturbance or further environmental change^45,46^.

**Figure 3:**
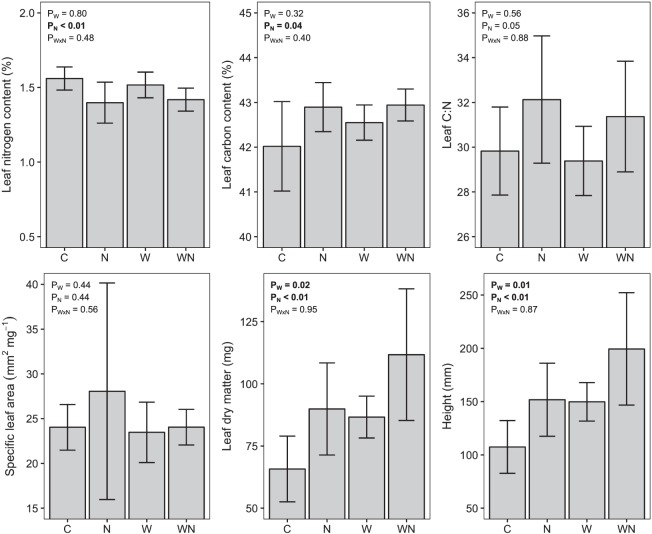
Colonizing plant traits in global change treatments. Bars are treatment means with one standard error. *P*-values resulting from two-way ANOVAs are listed in the upper left corner of each plot (values < 0.05 in bold; P_N_ is nitrogen main effect, P_W_ is warming main effect, and P_W × N_ is the warming by nitrogen interaction).

While N addition alone drove functional diversity responses of the colonizing plant community, both N addition and soil warming selected for univariate functional trait means that differed from control plots. The colonizing plant community was taller under both drivers (Fig. 3). This response suggests a shift toward conditions favoring competitive ability over tolerance of abiotic stress, in line with other warming studies^47^. Changes in whole community (abundance-weighted) leaf traits indicated a shift toward more conservative resource-use strategies for colonizing plant species caused by both N addition and soil warming^48^. Specifically, this entailed increasing leaf dry matter content in soil warming plots, and increasing leaf C and dry matter content with increasing N content in N addition plots (Fig. 3). The increases in plant height, leaf C and dry matter content of the colonizing plant community were positively correlated with percent cover of the planted tussock *C. rigida* (Table S6), suggesting that the increasing dominance of this species reduced resource availability and thus altered the functional trait distribution of the colonizing species. The decrease in leaf N content (LNC) in N addition plots differs from other studies, which have found that nutrient addition selects for higher nutrient content in leaf tissues^49^. However, the N applied in this study may have quickly been utilized by the dominant planted tussocks, as there was no change observed in the total soil N pool (Table S1). Congruent with this hypothesis, previous results from this experiment showed that N addition increased the N content of tussock leaves^38^, but not soil N^39^. Thus, the shifts in leaf traits of the colonizing species that we observed with N addition suggest selection for more resource conservative strategies in an increasingly competitive plant community where the dominant tussocks sequester resources, rather than a direct response to increased nutrient availability.

As with taxonomic composition, the functional characteristics or traits of soil bacterial communities (i.e. the relative abundance of functional gene categories) were significantly affected by soil warming (Fig. 1D, Table S3). The functional richness of the soil bacterial community, assessed by the number of unique gene categories detected, was significantly reduced in soil warming plots (Fig. 2D, Table S4). This reduction in functional richness, together with the lack of change in taxonomic richness, suggests that the warming treatment selected for soil bacteria with similar traits, thus increasing functional redundancy but reducing functional capacity of the belowground community. These community-level responses, observed as changes in the relative abundance of functional gene categories, did not depend on the level of the functional hierarchy considered (Fig. S1B, Table S7). Changes in the functional diversity of the soil bacterial community may have consequences for the ecosystem, as loss of functional diversity is often associated with decreasing ecosystem functioning^50,51^. In contrast, functional redundancy is often associated with stability, though the level of redundancy may depend on environmental context^52^.

Shifts in the relative abundance of functional gene categories revealed specific ecological characteristics that were selected for by the soil warming treatment. At the broadest level of functional gene categories, soil warming was associated with decreases in core metabolic functions associated with cell growth and turnover (i.e. RNA metabolism, nucleosides and nucleotides, and cell wall and capsule) (Fig. S4). This is indicative of selection for a slower growing, more resource-conservative soil bacterial community.

**Figure 4:**
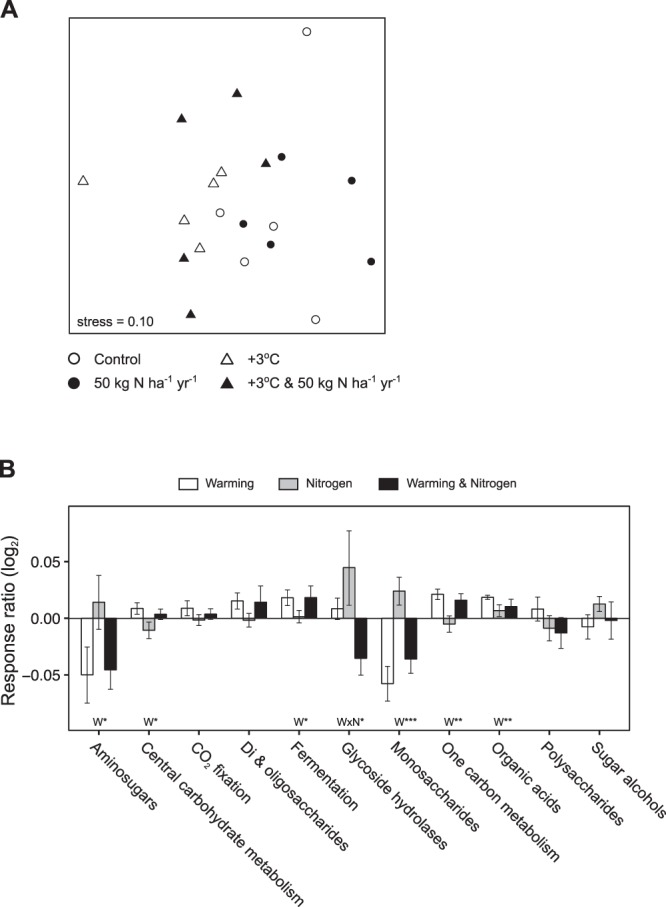
Shifts in the relative abundance of soil bacterial gene categories associated with carbon cycling in global change treatments. (**A**) Non-metric multidimensional scaling ordination among experimental plots based on Bray-Curtis dissimilarity calculated from the relative abundance of metagenome reads assigned to ‘Carbohydrate’ subsystems (categories at the 3^rd^ level of a hierarchy as defined in the SEED database^73^). Results of permanova analyses for these data are as follows; W (soil warming): *R*^2^ = 0.26, *p* < 0.001; N (nitrogen addition): *R*^2^ = 0.06, *p* = 0.16; W × N: *R*^*2*^ = 0.01, *p* = 0.93. (**B**) Response ratios (log_2_) of the relative abundance of metagenome reads assigned to ‘Carbohydrate’ subsystems (2^nd^ level of hierarchy as defined in the SEED database^73^). Bars represent the mean response ratio of treatment plots to control plots with one standard error. Significant treatment effects from linear models are indicated by N for nitrogen addition, W for soil warming, and W × N for the interactive effect. * indicates *p* < 0.05, ** indicates *p* < 0.01, and *** indicates *p* < 0.001.

To further characterize shifts in the functional capacity of the soil bacterial community in response to N addition and soil warming, we focused on functional gene categories associated with N and C cycling, which are likely to be involved in feedbacks between above and belowground communities^53^. Nitrogen addition had a minimal effect on either category (Figs S5, S6). In contrast, warming altered functional characteristics of the soil bacterial community associated with C cycling (Fig. 4A); we observed decreases in genes associated with aminosugar and monosaccharide metabolism, and increases in genes associated with fermentation, one-carbon and organic acid metabolism (Fig. 4B). There was also a decrease in gene categories associated with cellulolytic potential (the ability to break down more complex organic molecules such as cellulose and chitin) (Fig. S5). The influence of soil warming on genes associated with C cycling may be driven partly by tradeoffs among microbial traits. Stress tolerance (i.e. coping with low soil moisture in warmed plots) and cellulolytic potential are energetically expensive traits, and previous studies suggest that there are physiological tradeoffs between the two (i.e., cellulolytic potential is negatively correlated with drought tolerance)^54^. Moreover, these decreases in genes associated with metabolism of simple C molecules and broad shifts in functional genes toward a more oligotrophic soil bacterial community are similar to the early phases of belowground responses to warming recently observed in a long-term study of a temperate hardwood forest^24,29,55^.

Finally, we determined whether between-plot differences in taxonomic and trait composition were correlated above and belowground, and whether this correlation was weakened by the global change drivers. Between-plot differences in taxonomic composition of the soil bacterial community were not related to between-plot differences in the composition of the colonizing plant community for any combination of global change drivers, though this relationship was only marginally non-significant at *P* = 0.08 under control conditions (Fig. S7A). Functional capacity of the soil bacterial community was had the strongest correlation with the functional traits of the colonizing plant community when only the control plots were considered (*P* = 0.07, Fig. S7B). There were no significant relationships between the cover of the planted tussock community and any measure of taxonomic or functional diversity of the soil bacterial community (Table S6). These results are in line with other studies that found functional characteristics to be more effective than taxonomy in detecting congruence between above and belowground communities^56^. Most importantly, these changes show that the plant and soil microbial communities had distinct responses to the same global change drivers, as the correlations between these two components broke down under both experimental drivers. However, it is possible that there is a lag before changes in the plant community are realized belowground (and vice versa), and that this lag period extended beyond the four years of treatment measured in this study.

We found that after four years of experimental treatment, plant and soil bacterial communities responded very differently to two major global change drivers, both taxonomically and functionally. While the drivers that most strongly affected the aboveground and belowground community differed (i.e., N addition and warming, respectively), the general response was nevertheless comparable: plant and soil bacterial communities both moved toward more resource conservative strategies in response to their specific driver suggesting a general shift toward more competitive conditions with global change. Both communities occupied a smaller range of possible trait values, and exhibited changes in functional redundancy, which likely impacts their resilience to further environmental change. Plant communities shifted toward reduced redundancy, whereas redundancy increased in the soil microbial community. Moreover, the greater impact of N addition aboveground and warming belowground suggests that plant and soil bacterial communities have distinct tolerance ranges for these drivers. In conclusion, our results clearly show that whole-ecosystem responses cannot be predicted by looking only at subsystems in isolation. The absence of changes aboveground may belie major changes belowground, and vice versa, such that previous assessments may have underestimated the whole-system impact of global environmental changes.

## Materials and Methods

### Experimental design

To assess the ecosystem impacts of soil warming and nitrogen (N) addition, an experiment was established in January 2009 in native subalpine tussock grassland at the University of Canterbury Cass Mountain Research Area on the South Island of New Zealand (43.03° S, 171.75° E). Soils at the site are classified as acidic allophane brown following the New Zealand Soil Classification (Typic Dystrochrept by USDA). Vegetation and topsoil to 20 cm were removed from twenty 3.5 × 3.5 m plots to generate greater homogeneity of soil chemistry and to enable burial of heating and dummy cables. The two treatments (warming and N addition), each with two levels (elevated and control), were assigned to experimental plots in a factorial randomized design with five replicates per treatment combination. Plots were separated by 1 m borders. In each plot, electric heating cables (Argus Heating Ltd, Christchurch, New Zealand) or control dummy cables were installed and covered with original topsoil. Identical native tussock communities were then planted to resemble a semi-natural tussock grassland (our study system), rather than relying entirely on self-colonization, which could have produced a community dominated by pastoral weeds with high dispersal ability. These planted communities consisted of native New Zealand tussock grasses: *Festuca novae-zelandiae* (50 individuals per plot), *Poa cita* (50 per plot), *Chionochloa rigida* (22 per plot), and *Chionochloa flavescens* (12 per plot) (all herbaceous perennial tufted grasses). See Table S8 for traits of planted species obtained from the literature^57–59^. The four tussock species were chosen to represent characteristic species from across the region, but also those that were commercially available in large enough numbers for the experiment. Short-tussock grassland (which spread onto the Canterbury Plains following historical land clearing by burning) is dominated by *Festuca novae-zelandiae* and *Poa cita*^60^, so we planted these species at higher abundance. The higher-altitude forests on the inland ranges in Canterbury were replaced by shrubland and grasslands of *Chionochloa rigida*, which colonized from higher altitudes^60^. We included *Chionochloa flavescens* at the lowest abundance because it does not occupy a large extent of the region, though it dominates the zone 200 m above treeline on the wetter mountainous areas of the South Island^60^, so comprises an important component of the regional flora. Campbell CR1000 (Campbell Scientific, USA) data loggers recorded the temperature of thermocouples in each plot every minute. Each heated plot was paired with an unheated plot, and power was switched on in heated plots as necessary to maintain a 3 °C temperature difference above thermocouples in the unheated plot (slightly below the 3.5 °C mean increase predicted for this century under a high carbon scenario^61^). The N treatment consisted of five additions per year of 10 kg ha^−1^ N-NH_4_NO_3_ dissolved in 4 L of water and applied evenly over each plot. An equal volume of water was added to non-N addition control plots. This annual rate of 50 kg N ha^−1^ is equivalent to moderate global deposition rates^62^, and considerably exceeds the local background deposition of 6–8 kg N ha^−1^ year^−1^ total (including non-mineral forms) for the study area. Measured soil parameters (total soil C, N, and pH) were not impacted by the treatments (Table S1). However, previous research from this experiment revealed that soil warming and N addition additively increased soil respiration rates, and N addition reduced the heterotrophic fraction of soil respiration because only autotrophic (but not heterotrophic) soil respiration increased^39^.

### Plant community composition

Plant community composition and functional traits were assessed in late austral summer 2013 (i.e., 4 years after set-up). Cover of all plant species present in each experimental plot (planted tussocks and colonizing species) was assessed using five 1 × 1 m quadrats. Quadrats were placed at least 20 cm from the edge of plots to avoid edge effects. A modified Braun-Blanquet scale^63^ was used to estimate the mean percentage cover score for each vascular plant species in each quadrat. Cover classes used were <0.1%, 0.1–0.9%, 1–5%, 5–25%, 26–50%, 51–75%, and 75–100%. Overlapping vegetation of more than one species was attributed to all species in percent cover estimates. The cover class medians for each species in each of the five quadrats within each plot were averaged to estimate the mean absolute percentage cover scores per plot. The percent cover of any extra species present in the plot, but not present in any quadrat from that plot, was estimated separately. This approach ensured that rare species were included in the analysis and that species richness was accurately assessed at the plot level.

### Plant functional traits

Vegetative traits, indicative of plant resource economic strategy and associated with ecosystem processes, were measured according to standard protocols^43^ for plant species that colonized the plots. Plant height and leaf traits were measured for five individuals per species (or all individuals if there were fewer than five in the whole plot). Only the rarest species across the experiment were excluded from trait measurements, so that species with measured traits made up more than 93% of the total colonizing species cover in each plot. Traits were not measured for the planted tussock species, as their establishment was not related to the experimental treatments, and they were therefore not subject to the same degree of environmental filtering as were the colonizing species. However, we analysed separately their response to the different treatments and used this as a predictor in analyses of above and belowground communities (see below). Vegetative plant height was measured from the ground to the tip of the highest vegetative organ. Leaf area (LA), specific leaf area (SLA) and leaf dry matter content (LDMC) were measured for two mature leaves from each individual plant. When sufficient dry leaf biomass was available, leaf nitrogen content (LNC) and carbon content (LCC) were measured for composite samples of the two leaves collected from each plant. If dry leaf biomass was too low for nutrient concentration measurements, composite samples were generated by pooling leaves from individuals of the same species collected from the same experimental plot. Leaf samples were dried at 60 °C, ground and analysed on a LECO CNS-2000 Elemental Analyser (LECO Australia Pty Ltd., Sydney, Australia).

To determine how plant functional traits responded to the experimental treatments at the community level, we calculated community weighted means (CWM) for each trait. Trait measurements were first averaged by species for each experimental plot. To calculate the CWM trait values for an experimental plot, the treatment mean traits were scaled by the relative cover of the colonizing species present in the plot. This approach accounted for both shifts in plant community composition and intraspecific trait variation (i.e. trait variation among individuals of the same species from different plots exposed to soil warming and N addition) in the calculation of CWMs. Incorporating intraspecific variation in traits is important because it contributes significantly to a range of ecological processes^64,65^.

### Soil metagenomes

In May 2013, four soil cores (2 cm diameter and 10 cm depth) were collected per plot. Cores were taken at least 20 cm from the edge of plots to avoid edge effects. The soil cores were pooled, sieved (2 mm) and three replicate subsamples of 2 g of soil were stored at −20 °C in LifeGuard^TM^ Soil Preservation Solution (Mo Bio Laboratories, CA, USA). Total community DNA was extracted from each subsample with the Mo Bio RNA PowerSoil® Total RNA Isolation kit followed by the RNA PowerSoil® DNA Elution Accessory kit (RNA yields were insufficient for metatranscriptome sequencing, so only shotgun metagenomes were generated). The three replicate DNA extractions from each plot were pooled and purified with the Mo Bio PowerClean® DNA Clean-Up kit. Total DNA was shipped to Macrogen Inc. (Seoul, Republic of Korea) for library preparation and sequencing of 2 × 100 PE shotgun reads on the Illumina HiSeq 2000 platform multiplexing 6 samples per lane. Prior to analysis, all reads were cleaned using the AdapterRemoval tool^66,67^. Shotgun reads were submitted to the MG-RAST web server for annotation^68^. Sequencing and annotation statistics are provided in Table S9. MG-RAST splits the dataset into reads belonging to 16S rRNA gene sequences (used to generate taxonomic profiles) and reads associated with protein-coding genes, which are used to generate functional profiles. Taxonomic profiles were generated from 16S annotations compared to the M5rna database, which integrates the SILVA^69^, GreenGenes^70^ and RDP^71^ rRNA databases, using a maximum e-value of 1E-5, a minimum identity of 60%, and a minimum alignment length of 15. Functional profiles were generated from protein-coding annotations compared to the M5nr database^72^ using a maximum e-value of 1E-5, a minimum identity of 60%, and a minimum alignment length of 15, and viewed in the SEED subsystems functional hierarchy^73^. Sequence coverage plots are presented in Fig. S8. In this annotation framework, ‘functional roles’ are grouped into ‘subsystems’ (for example, a subsystem may be the collection of functional roles that make up a metabolic pathway). These subsystems are then grouped into broader categories that form a 4-level hierarchy. Community-aggregated traits of the soil microbial community can be inferred from these subsystems^35^, providing a means for estimating (within the limits of available annotated genomes) soil microbial community responses to global change drivers from a functional perspective. Archaea made up less than 1% of soil microbial taxonomic profiles, and no treatment responses were observed (Fig. S9), so microbial responses examined here comprise primarily soil bacteria.

Treatment bias in the number of 16S or functional reads that could be annotated could in theory lead to misinterpretation of results. To ensure no such bias was present in either the taxonomic or functional data set, we ran generalized linear models with a Poisson error distribution to test whether the number of bacterial 16S rRNA and protein-coding reads differed across treatments. Models were fitted using quasi-likelihood, because there was evidence of overdispersion in both cases. These tests confirmed that the experimental treatments did not significantly impact the number of 16S reads (warming F_1,16_ = 0.81, p = 0.38; nitrogen F_1,16_ = 0.07, p = 0.80, warming × nitrogen F_1,16_ = 0.67, p = 0.43) or protein-coding reads (warming F_1,16_ = 2.63, p = 0.12; nitrogen F_1,16_ = 0.33, p = 0.57, warming × nitrogen F_1,16_ = 0.29, p = 0.60) that could be annotated.

### Soil parameters

Soil moisture measurements to 10 cm depth (Theta Probe ML2, DeltaT Devices, Cambridge, UK) were taken immediately prior to soil core collection. Total soil carbon and nitrogen were measured from subsamples of sieved soil with a LECO CNS-2000 Elemental Analyser on some of the remaining sieved soil collected for DNA extraction. Soil pH was measured in a 1:2.5 soil to water slurry.

### Statistical analyses

All analyses were performed in the R environment^74^. We assessed the impact of global change drivers on diversity (univariate) and composition (multivariate) measures from taxonomic and trait measures for plant and soil bacterial communities (see Table S2). For the colonizing plant community, functional diversity indices^44,75^ were calculated from CWM trait values using the distance-based framework implemented in the ‘FD’ package^76^ for R. All other diversity measures (number of plant species and bacterial taxa, functional capacity of the soil bacterial community, Shannon (H) and Inverse Simpson indices) were assessed with the ‘vegan’ R package^77^. To ensure that soil microbial community responses were not specific to a particular taxonomic level, we assessed taxonomic responses for both bacterial phyla and genera, and functional capacity at the most specific (functional roles) and most general levels (level 1 subsystems) of the SEED hierarchy.

To assess composition responses, Bray-Curtis dissimilarity between experimental plots was calculated from the multivariate datasets. Non-metric multidimensional scaling (NMDS) ordinations were generated to visualize relationships among experimental plots. These distance matrices were then analysed in two-way permutational multivariate analyses of variance (PERMANOVA)^78,79^ to test for impacts of soil warming, N addition and their interaction on community composition from both taxonomic and functional perspectives. PERMANOVA tests were conducted with the ‘adonis’ function in the ‘vegan’ R package and 999 permutations of the data. To assist in interpretation of these tests, univariate analyses were undertaken on each of the variables comprising the multivariate responses.

To assess trait diversity of the colonizing plant community and other univariate responses to soil warming, N addition, and their interaction, we conducted general linear models. For continuous responses (i.e. soil pH and C:N, Shannon and Inverse Simpson diversity indices, individual CWM traits), the data were transformed (BoxCox) when assumptions regarding normality and homogeneity of variance were not met. Responses that were expressed as percentages (i.e. soil moisture, total soil C and N, plant cover, relative abundance of bacterial taxa and SEED subsystems) were logit transformed^80^. For count data (i.e. number of colonizing plant species, taxonomic richness and functional capacity of soil microbial communities), generalized linear models with Poisson error distributions were run to test for treatment impacts. Statistical significance of treatments was determined with analysis of deviance tests.

Procrustes rotation and permutation tests were run to determine whether soil warming and/or N addition impacted the ability to predict among-plot relationships in soil microbial composition from among-plot relationships in colonizing plant composition or vice versa. The relationships considered were plant taxonomy: soil microbial taxonomy, and plant traits: soil microbial traits. For each experimental treatment combination, we separately assessed whether the strength and significance of these relationships was impacted by soil warming and N addition, individually and together.

## Supplementary information

Supplementary information for <i>Above and belowground community strategies respond to different global change drivers</i>

## Data Availability

Raw sequence data for the metagenomes will be made publicly available upon acceptance (MG-RAST IDs: 4570855.3–4570874.3). All other data is available by request to the corresponding authors.
