# Supplementary material for: Above and belowground community strategies respond to different global change drivers
Source: Sci Rep. 2019 Feb 22;9:2540. doi: 10.1038/s41598-019-39033-4 (PMC6385336; doi:10.1038/s41598-019-39033-4)

**Supplementary information for *Above and belowground community strategies respond to different global change drivers***

Karen L. Adair[1,2], Stinus Lindgreen[1,3], Anthony M. Poole[1,4], Laura M. Young[1], Maud Bernard-Verdier[5,6], David A. Wardle[7,8], Jason M. Tylianakis[1,9]

[1] School of Biological Sciences, University of Canterbury, Private Bag 4800, Christchurch 8140, New Zealand

[2] Present address: Department of Entomology, Comstock Hall, Cornell University, Ithaca, NY, USA 14853

[3] Present address: H. Lundbeck A/S, Ottiliavej 9, 2500 Valby, Denmark

[4] Present address: School of Biological Sciences, University of Auckland, Private Bag 92019, Auckland 1142, New Zealand

[5] Bio-Protection Research Centre, Lincoln University, PO Box 85084, Lincoln 7647, Canterbury, New Zealand

[6] Present address: Freie Universität Berlin, Institut für Biologie, Königin-Luise-Str. 1-3, 14195 Berlin-Dahlem, Germany

[7] Department of Forest Ecology and Management, Swedish University of Agricultural Sciences, SE901-83 Umea, Sweden

[8] Asian School of the Environment, Nanyang Technological University, 50 Nanyang Avenue, Singapore 639798

[9] Department of Life Sciences, Imperial College London, Silwood Park Campus, Buckhurst Road, Ascot, Berkshire SL5 7PY, United Kingdom

**Corresponding authors:**
Karen L. Adair. Email: kla89@cornell.edu.
Jason M. Tylianakis. Email: jason.tylianakis@canterbury.ac.nz, Ph: +64 3 369 5379.

**Table S1 –** Mean soil properties for global change treatment plots (W: warming, N: nitrogen addition, WxN: warming and nitrogen) with one standard deviation listed in parentheses. For soil pH, carbon (C), nitrogen (N), and C:N, n = 2 and n = 3 for the WxN and warming treatments respectively; n = 5 for all other cases. *P*-values from analysis of deviance tests for impacts of warming, N addition and their interaction are given in the last three columns with values $< 0.05$ in bold. An earlier assessment (after three years), but including all replicates (n = 5) also found no differences in total N or total C among plots (Graham *et al.*, 2014).

| | | | | | *p* - values | | |
|---|---|---|---|---|---|---|---|
| | Control | Warming (W) | Nitrogen (N) | W x N | W | N | N x W |
| Moisture (%) | 32.9 (2.74) | 26.7 (2.82) | 32.5 (4.46) | 25.7 (4.85) | **<0.01** | 0.64 | 0.82 |
| pH | 5.35 (0.06) | 5.30 (0.06) | 5.35 (0.07) | 5.39 (0.06) | 0.60 | 0.40 | 0.27 |
| Carbon (%) | 4.12 (0.24) | 4.14 (0.08) | 4.31 (0.16) | 4.27 (0.13) | 0.83 | 0.11 | 0.74 |
| Nitrogen (%) | 0.32 (0.02) | 0.31 (0.02) | 0.33 (0.02) | 0.33 (0.01) | 0.45 | 0.34 | 0.86 |
| C:N | 12.9 (0.29) | 13.2 (0.40) | 13.0 (0.25) | 13.1 (0.28) | 0.19 | 0.63 | 0.37 |

**Table S2 -** Details of taxonomic and trait response variables for plant and soil microbial communities. For the soil microbial communities, functional roles (i.e. annotated protein function) and level 4 subsystems refer to the most specific and broadest levels respectively of the functional hierarchy used to annotate microbial protein-coding genes (Wilke *et al.*, 2012; Overbeek *et al.*, 2005).

| | Plants | Soil Microbes |
|---|---|---|
| **Taxonomic responses** | | |
| Diversity | Number of colonizing plant species. Shannon and inverse Simpson diversity indices for colonizing plant community. | Number of bacterial genera and bacterial phyla detected. Shannon and inverse Simpson diversity indices for relative abundance of bacterial genera and phyla. |
| Composition | Percent cover of colonizing species. | Relative abundance of bacterial genera and phyla. |
| **Trait responses** | | |
| Diversity | Functional richness, evenness, divergence, and dispersion of the colonizing plant species (Villéger *et al.*, 2008; Laliberté and Legendre, 2010). | Number of functional roles and level 4 subsystems detected. Shannon and inverse Simpson diversity indices for relative abundance of functional roles and level 4 subsystems. |
| Composition | Standardized community weighted mean trait values of the colonizing plant species. | Relative abundance of functional roles and level 4 subsystems |

**Table S3 -** Permutational multivariate analysis of variance (permanova) results based on plant taxonomy (percent cover of colonizing plant species), plant traits (community weighted mean trait values of the colonizing plant species), soil microbial taxonomy (relative abundance of bacterial phyla), and soil microbial traits (relative abundance of microbial functional roles, the most specific level of the functional hierarchy (Overbeek *et al.*, 2005)). Partial $R^2$ values are given with *p*-values listed in parentheses (values < 0.05 in bold; W: warming, N: nitrogen addition).

| | Plants | Soil microbes |
|---|---|---|
| Taxonomy | W: 0.03 (0.71)<br>N: 0.06 (0.37)<br>WxN: 0.04 (0.56) | **W: 0.31 (<0.001)**<br>N: 0.07 (0.10)<br>WxN: 0.04 (0.33) |
| Traits | **W: 0.22 (0.007)**<br>**N: 0.25 (0.005)**<br>WxN: 0.02 (0.29) | **W: 0.13 (0.02)**<br>N: 0.07 (0.23)<br>WxN: 0.02 (0.87) |

**Table S4** - Mean diversity metrics for plant and soil microbial communities in global change treatment plots (W: warming, N: nitrogen addition) with one standard deviation listed in parentheses. For the soil microbial communities, functional roles (i.e. annotated protein function) and level 4 subsystems refer to the most specific and broadest levels respectively of the functional hierarchy used to annotate microbial protein-coding genes (Wilke *et al.*, 2012; Overbeek *et al.*, 2005). *P*-values from analysis of deviance tests for impacts of warming, N addition and their interaction are given in the last three columns with values < 0.05 in bold. A large number of hypothesis tests in each category (e.g. three measures of plant diversity, tested against three treatment effects) inflates the type I error rate. To account for this, $P_{combined}$ is the probability of achieving the number of significant values observed, at or below the observed alpha, given the number of tests (Moran, 2003).

| | Control | Warming (W) | Nitrogen (N) | W x N | *p* - values | | |
|---|---|---|---|---|---|---|---|
| | | | | | W | N | W x N |
| **Plant Taxonomic Diversity** ($P_{combined}$=0.212) | | | | | | | |
| Colonizing species richness | 12.8 (2.05) | 13.4 (2.61) | 11.8 (1.10) | 10.0 (2.24) | 0.53 | **0.03** | 0.21 |
| Shannon diversity (H) | 1.45 (0.15) | 1.50 (0.24) | 1.36 (0.37) | 1.12 (0.45) | 0.54 | 0.13 | 0.33 |
| Inverse Simpson index (1/D) | 2.93 (0.50) | 3.10 (0.96) | 3.01 (1.39) | 2.29 (1.01) | 0.37 | 0.43 | 0.34 |
| *Plant functional groups* ($P_{combined}$=0.108) | | | | | | | |
| Nitrogen-fixing species richness | 1.8 (0.84) | 2.4 (1.14) | 1.2 (0.45) | 1.2 (0.45) | 0.60 | 0.11 | 0.69 |
| Cover of nitrogen-fixing species | 5.49 (4.18) | 8.16 (12.77) | 1.14 (1.58) | 0.67 (0.45) | 0.92 | **0.02** | 0.66 |
| **Plant Trait Diversity** ($P_{combined}$=0.24) | | | | | | | |
| Functional richness | 1.65 (1.05) | 1.39 (0.91) | 0.57 (0.35) | 0.69 (0.32) | 0.83 | **0.02** | 0.58 |
| Functional evenness | 0.51 (0.20) | 0.53 (0.10) | 0.65 (0.15) | 0.61 (0.19) | 0.90 | 0.15 | 0.74 |
| Functional divergence | 0.86 (0.10) | 0.82 (0.19) | 0.88 (0.09) | 0.79 (0.23) | 0.40 | 0.99 | 0.76 |
| Functional dispersion | 1.76 (0.39) | 1.63 (0.36) | 1.70 (0.61) | 1.45 (0.72) | 0.44 | 0.64 | 0.80 |
| **Soil Microbial Taxonomic Diversity** | | | | | | | |
| *Bacterial phyla* ($P_{combined}$=0.002) | | | | | | | |
| Richness | 26.0 (0.71) | 25.6 (0.55) | 25.4 (0.55) | 25.4 (0.89) | 0.93 | 0.86 | 0.93 |
| Shannon diversity (H) | 1.95 (0.02) | 1.97 (0.02) | 1.93 (0.02) | 1.94 (0.01) | 0.15 | **0.03** | 0.63 |
| Inverse Simpson index (1/D) | 4.80 (0.12) | 5.08 (0.21) | 4.73 (0.11) | 4.87 (0.05) | **0.004** | **0.03** | 0.27 |
| *Bacterial genera* | | | | | | | |
| Richness | 597 (14.7) | 578 (20.9) | 594 (12.4) | 582 (20.2) | 0.16 | 0.99 | 0.76 |
| Shannon diversity (H) | 5.03 (0.04) | 4.98 (0.07) | 5.03 (0.05) | 5.03 (0.04) | 0.25 | 0.28 | 0.22 |

| | | | | | | | |
|---|---|---|---|---|---|---|---|
| Inverse Simpson index (1/D) | 50.9 (3.88) | 46.6 (4.55) | 51.0 (4.88) | 51.3 (3.43) | 0.30 | 0.22 | 0.24 |
| **Soil Microbial Trait Diversity** | | | | | | | |
| *SEED L1 subsystems* | | | | | | | |
| Richness | 28 (0) | 28 (0) | 28 (0) | 28 (0) | - | - | - |
| Shannon diversity (H) | 2.80 (0.002) | 2.80 (0.002) | 2.80 (0.002) | 2.80 (0.002) | 0.17 | 0.15 | 0.99 |
| Inverse Simpson index (1/D) | 12.2 (0.03) | 12.2 (0.03) | 12.2 (0.03) | 12.2 (0.05) | 0.12 | 0.24 | 0.93 |
| *Functional roles (SEED L4)* ($P_{combined}$= 8.8 x $10^{-5}$) | | | | | | | |
| Richness | 6494 (57) | 6383 (166) | 6579 (123) | 6419 (163) | **0.02** | 0.31 | 0.69 |
| Shannon diversity (H) | 6.91 (0.005) | 6.90 (0.013) | 6.92 (0.012) | 6.91 (0.009) | **0.007** | **0.03** | 0.98 |
| Inverse Simpson index (1/D) | 430 (3.51) | 422 (3.04) | 434 (5.10) | 425 (4.26) | **<0.001** | 0.08 | 0.66 |

**Table S5 –** Mean absolute percent cover estimates of planted tussock species, colonizing plant species, and plant functional groups with one standard error listed in parentheses. Overlapping vegetation of more than one species was attributed to all species in percent cover estimates. Species that were not detected in any plot for a particular treatment combination are listed as *n.d.* *P*-values from two-way ANOVAs for impacts of warming, N addition and their interaction are given in the last three columns with values < 0.05 in bold. Note that these tests are intended only to assist with interpreting the multivariate analyses presented in Figure 1A and Table S4, and the individual *p*-values presented here should be interpreted with caution, as the large number of tests conducted would lead to an inflated probability of type I error. Nevertheless, the probability of observing 7 significant tests (at an alpha of 0.05) out of 69 is p = 0.035 (Moran, 2003).

* *indicates species that form N-fixing associations*
† *indicates native species*

| | | | | | | *p* - values | | |
|---|---|---|---|---|---|---|---|---|
| | Functional group | Control | Warming (W) | Nitrogen (N) | W&N | W | N | W x N |
| **Planted Tussock Species** | | | | | | | | |
| *Chionochloa rigida*† | grass | 30 (3.9) | 38 (5.8) | 40 (2.3) | 55 (1.8) | **$p < 0.05$** | **$p < 0.01$** | n.s. |
| *Poa cita*† | grass | 27 (5.2) | 23 (2.5) | 26 (4.3) | 29 (3.7) | n.s. | n.s. | n.s. |
| *Chionochloa flavescens*† | grass | 5 (0.6) | 0.5 (0.2) | 1 (0.8) | 0.5 (0.2) | **$p < 0.01$** | **$p < 0.001$** | **$p < 0.01$** |
| *Festuca novaezelandiae*† | grass | 14 (1.1) | 12 (2.8) | 10 (2.1) | 7 (2.3) | n.s. | $p < 0.1$ | n.s. |
| **Colonizing Species** | | | | | | | | |
| *Achillea millefolium* | dicotyledonous herb | 0.04 (0.02) | 0.15 (0.11) | 0.76 (0.73) | 0.15 (0.14) | n.s. | n.s. | n.s. |
| *Agrostis capillaris* | grass | 12.1 (3.7) | 12.2 (3.1) | 16.6 (3.8) | 16.6 (6.8) | n.s. | n.s. | n.s. |
| *Anthoxanthum odoratum* | grass | 2.31 (1.26) | 2.16 (0.76) | 3.85 (1.75) | 2.50 (1.37) | n.s. | n.s. | n.s. |
| *Carex breviculmis*† | sedge | 0.002 (0.002) | n.d. | n.d. | n.d. | | | |
| *Cerastium fontanum* | dicotyledonous herb | 0.08 (0.03) | 0.16 (0.12) | 0.21 (0.15) | 0.02 (0.02) | n.s. | n.s. | n.s. |
| *Crepis capillaris* | rosette dicot | 0.67 (0.24) | 0.79 (0.46) | 0.73 (0.28) | 0.40 (0.38) | n.s. | n.s. | n.s. |
| *Cytisus scoparius** | legume | n.d. | 0.14 (0.14) | n.d. | n.d. | | | |

| | | | | | | | | |
|---|---|---|---|---|---|---|---|---|
| *Dactylis glomerata* | grass | n.d. | 0.01 (0.01) | n.d. | 0.01 (0.01) | n.s. | n.s. | n.s. |
| *Discaria toumatou*†* | non-legume N fixer | 0.02 (0.02) | 0.01 (0.01) | n.d. | 0.01 (0.01) | n.s. | n.s. | n.s. |
| *Festuca rubra* | grass | 0.02 (0.02) | 0.02 (0.02) | 0.12 (0.12) | 0.60 (0.60) | n.s. | n.s. | n.s. |
| *Veronica brachysiphon*† | shrub | n.d. | n.d. | 0.002 (0.002) | n.d. | | | |
| *Pilosella officinarum* | rosette dicot | 1.28 (0.35) | 1.27 (0.77) | 2.12 (0.70) | 0.83 (0.33) | n.s. | n.s. | n.s. |
| *Pilosella praelta* | rosette dicot | 0.33 (0.16) | 0.88 (0.60) | 1.01 (0.67) | 0.17 (0.11) | n.s. | n.s. | n.s. |
| *Holcus lanatus* | grass | 0.20 (0.10) | 0.20 (0.13) | 0.06 (0.05) | 0.02 (0.02) | n.s. | $p < 0.1$ | n.s. |
| *Hypochaeris radicata* | rosette dicot | 0.47 (0.14) | 1.66 (0.95) | 1.96 (0.90) | 1.00 (0.42) | n.s. | n.s. | n.s. |
| *Leptospermum scoparium*† | small tree | n.d. | 0.002 (0.002) | n.d. | n.d. | | | |
| *Linum catharticum* | dicotyledonous herb | 0.04 (0.04) | 0.27 (0.23) | 0.002 (0.002) | n.d. | n.s. | n.s. | n.s. |
| *Lotus pedunculatus** | legume | n.d. | 2.65 (2.64) | n.d. | n.d. | | | |
| *Luzula rufa*† | rush | 0.06 (0.04) | 0.03 (0.02) | 0.02 (0.02) | n.d. | n.s. | $p < 0.1$ | n.s. |
| *Ozothamnus leptophyllus*† | shrub | n.d. | 0.002 (0.002) | n.d. | n.d. | | | |
| *Pimelea oreophila*† | shrub | 0.02 (0.01) | 0.04 (0.02) | 0.01 (0.003) | 0.004 (0.002) | n.s. | $\mathbf{p < 0.05}$ | n.s. |
| *Plantago lanceolata* | rosette dicot | n.d. | 0.002 (0.002) | 0.003 (0.003) | n.d. | n.s. | n.s. | n.s. |
| *Rumex acetosella* | dicotyledonous herb | 0.71 (0.21) | 0.43 (0.29) | 0.49 (0.35) | 0.56 (0.37) | n.s. | n.s. | n.s. |
| *Senecio wairauensis*† | dicotyledonous herb | n.d. | n.d. | 0.02 (0.02) | n.d. | | | |
| *Trifolium dubium** | legume | 0.04 (0.04) | 0.09 (0.05) | n.d. | n.d. | n.s. | $p < 0.1$ | n.s. |
| *Trifolium repens** | legume | 5.38 (1.87) | 5.27 (2.97) | 1.13 (0.70) | 0.66 (0.20) | n.s. | $\mathbf{p < 0.05}$ | n.s. |
| *Ulex europaeus** | shrub | n.d. | n.d. | 0.01 (0.01) | n.d. | | | |
| *Verbascum thapsus* | dicotyledonous herb | n.d. | n.d. | 0.02 (0.02) | n.d. | | | |
| *Vicia sativa** | legume | 0.06 (0.06) | n.d. | n.d. | n.d. | | | |
| | | | | | | | | |
| **Cover by functional group:** | | | | | | | | |
| Dicotyledonous herbs | | 0.86 (0.21) | 1.01 (0.42) | 1.50 (0.68) | 0.73 (0.33) | n.s. | n.s. | n.s. |
| Grasses | | 90.84 (9.70) | 87.22 (13.43) | 97.63 (6.29) | 111.33 (6.85) | n.s. | n.s. | n.s. |
| Legumes | | 5.47 (1.86) | 8.15 (5.71) | 1.13 (0.70) | 0.66 (0.20) | n.s. | n.s. | n.s. |
| Non-legume N-fixers | | 0.02 (0.02) | 0.01 (0.01) | 0.00 (0.00) | 0.01 (0.01) | n.s. | $p < 0.1$ | n.s. |

| | | | | | | | |
|---|---|---|---|---|---|---|---|
| Rosette dicots | 2.76 (0.71) | 4.60 (1.85) | 5.83 (1.45) | 2.40 (0.87) | n.s. | n.s. | $p < 0.1$ |
| Rushes | 0.06 (0.04) | 0.03 (0.02) | 0.02 (0.02) | 0.00 (0.00) | n.s. | n.s. | n.s. |
| Sedge | 0.002 (0.002) | 0.00 (0.00) | 0.00 (0.00) | 0.00 (0.00) | n.s. | n.s. | n.s. |
| Shrubs | 0.02 (0.01) | 0.04 (0.02) | 0.02 (0.01) | 0.004 (0.002) | n.s. | n.s. | $p < 0.05$ |
| Small trees | 0.00 (0.00) | 0.002 (0.002) | 0.00 (0.00) | 0.00 (0.00) | n.s. | n.s. | n.s. |

**Table S6 –** Relationships between variation in the planted tussocks (% cover of *C. rigida* and total percent cover of planted tussocks) and variation in the colonizing plant and soil bacterial community. Values are Pearson's correlation coefficients with *p*-values given in parentheses (18 degrees of freedom for all tests). Correlations with $p < 0.05$ are in bold.

| | % cover of *C. rigida* | Total % cover of the 4 planted tussock species |
|---|---|---|
| *Colonizing Plants* | | |
| Number of species | -0.28 (0.23) | -0.10 (0.68) |
| Shannon diversity | -0.17 (0.46) | 0.04 (0.88) |
| Inverse Simpson Index | -0.05 (0.84) | 0.04 (0.88) |
| Functional richness | -0.05 (0.85) | 0.38 (0.10) |
| Functional evenness | 0.37 (0.10) | 0.19 (0.41) |
| Functional divergence | -0.11 (0.64) | -0.03 (0.91) |
| Functional dispersion | -0.11 (0.65) | -0.03 (0.90) |
| Leaf nitrogen (%) [a] | -0.33 (0.15) | -0.04 (0.87) |
| Leaf carbon (%) [a] | **0.56 (<0.01)** | **0.50 (0.03)** |
| Leaf carbon to nitrogen [a] | 0.28 (0.23) | 0.11 (0.63) |
| Specific leaf area ($mm^2$ $mg^{-1}$) [a] | -0.01 (0.97) | 0.04 (0.85) |
| Leaf dry matter content (mg) [a] | **0.64 (<0.01)** | 0.36 (0.12) |
| Vegetative height (mm) [a] | **0.65 (<0.01)** | 0.36 (0.12) |
| | | |
| *Soil Bacterial Community* | | |
| Number of phyla | -0.09 (0.71) | 0.11 (0.66) |
| Number of genera | -0.32 (0.17) | -0.38 (0.10) |
| Shannon index (phyla) | -0.13 (0.57) | -0.14 (0.57) |
| Shannon index (genera) | 0.07 (0.78) | -0.11 (0.65) |
| Inverse Simpson index (phyla) | 0.05 (0.83) | 0.03 (0.89) |
| Inverse Simpson index (genera) | 0.12 (0.62) | -0.19 (0.42) |
| Number of L1 subsystems | n/a | n/a |
| Number of L4 subsystems | -0.28 (0.24) | -0.33 (0.15) |
| Shannon index (L1) | -0.14 (0.56) | -0.09 (0.70) |
| Shannon index (L4) | 0.06 (0.80) | 0.04 (0.86) |
| Inverse Simpson index (L1) | -0.06 (0.79) | -0.04 (0.86) |
| Inverse Simpson index (L4) | 0.11 (0.66) | 0.11 (0.53) |

[a] community weighted mean

**Table S7 -** Permutational multivariate analysis of variance results based on soil microbial taxonomy (relative abundance of bacterial genera), and soil microbial traits (relative abundance of functional roles, the most specific level of the subsystem hierarchy of microbial protein-coding genes). Partial $R^2$ values are given with *p*-values listed in parentheses (W: warming, N: nitrogen addition).

| | Soil microbes |
|---|---|
| Taxonomy | **W: 0.11 (<0.001)**<br>N: 0.05 (0.32)<br>WxN: 0.04 (0.62) |
| Traits | **W: 0.13 (0.002)**<br>N: 0.06 (0.23)<br>WxN: 0.04 (0.62) |

**Table S8 –** Traits of planted tussock species obtained from the literature. The total area covered by each planted tussock species in each experimental plot was estimated relative to the ground area and included cover that may have overlapped with other species. Treatment means for these plot area estimates are given with standard deviations in parentheses.

| | *Chionochloa rigida* | *Poa cita* | *Chionochloa flavescens* | *Festuca novae-zelandiae* |
|---|---|---|---|---|
| Max mean height (m) [a] | 1.50 | 1.00 | 1.50 | 1.00 |
| Leaf form[a] | Flat | Folded | Flat | Rolled |
| Max mean leaf length (mm) [a] | 800 | 400 | not available | 250 |
| Leaf width (mm) [a] | 7 | 2 | not available | 1 |
| Leaf carbon content (%) [b] | 43.8 | 43.5 | not available | 42.2 |
| Leaf nitrogen content (%) | 0.85 [b] | 0.99 [b] | 0.83 [c] | 0.98 [b] |
| | | | | |
| Cover area ($m^2$) [d] | | | | |
| Control | 3.7 (1.07) | 3.3 (1.42) | 0.6 (0.17) | 1.7 (0.31) |
| Warming | 4.6 (1.58) | 2.8 (0.69) | 0.1 (0.06) | 1.5 (0.77) |
| Nitrogen | 4.9 (0.64) | 3.2 (1.17) | 0.1 (0.21) | 1.2 (0.57) |
| Warming and nitrogen | 6.8 (0.50) | 3.5 (1.02) | 0.1 (0.06) | 0.9 (0.62) |

[a] from Ecological Traits of New Zealand Flora database (http://ecotraits.landcareresearch.co.nz/)
[b] from Craine *et al.*, 2005 acquired from TRY database (Kattge *et al.*, 2011)
[c] from Kerkhoff *et al.*, 2006 acquired from TRY database (Kattge *et al.*, 2011)
[d] total area cover of planted tussock species

**Table S9 –** Sequencing and annotation summary information for all individual plots. The + and - symbols in each treatment column indicate elevated (+) N or temperature vs. controls (-).

| Plot | Warming | Nitrogen | Read pairs | Predicted feature | Annotated protein | Predicted rRNA | Identified rRNA | # of species |
|---|---|---|---|---|---|---|---|---|
| **1** | **+** | **-** | 29,611,743 | 46,484,131 | 9,839,390 | 7,327,567 | 19,413 | 1377 |
| **2** | **-** | **-** | 30,858,156 | 50,121,979 | 10,835,089 | 7,442,500 | 18,424 | 1346 |
| **3** | **-** | **+** | 35,151,010 | 55,015,870 | 12,268,152 | 8,147,351 | 21,440 | 1434 |
| **4** | **+** | **+** | 27,033,253 | 41,402,700 | 8,910,670 | 6,626,905 | 15,886 | 1258 |
| **5** | **+** | **-** | 29,333,060 | 45,516,549 | 9,566,571 | 7,305,819 | 18,156 | 1334 |
| **6** | **-** | **+** | 29,956,983 | 48,329,419 | 10,601,195 | 7,236,463 | 18,858 | 1409 |
| **7** | **+** | **-** | 21,673,161 | 34,773,200 | 7,175,703 | 5,408,803 | 13,393 | 1147 |
| **8** | **+** | **+** | 26,348,400 | 42,974,861 | 8,963,950 | 6,789,656 | 16,816 | 1301 |
| **9** | **-** | **-** | 23,146,432 | 36,837,082 | 7,731,286 | 5,533,486 | 15,627 | 1257 |
| **10** | **-** | **+** | 28,931,475 | 42,155,534 | 8,791,476 | 7,140,310 | 18,303 | 1359 |
| **11** | **+** | **+** | 28,792,520 | 46,110,265 | 9,840,401 | 7,245,030 | 18,293 | 1377 |
| **12** | **-** | **+** | 31,418,120 | 49,487,033 | 10,494,762 | 7,674,382 | 18,052 | 1330 |
| **13** | **+** | **-** | 30,594,133 | 49,089,351 | 10,389,612 | 7,735,258 | 19,926 | 1386 |
| **14** | **+** | **-** | 26,683,544 | 47,364,072 | 8,427,486 | 6,522,775 | 15,105 | 1238 |
| **15** | **-** | **-** | 36,990,467 | 57,141,144 | 12,059,232 | 9,057,235 | 23,967 | 1480 |
| **16** | **-** | **-** | 30,199,988 | 49,594,683 | 10,433,117 | 7,424,290 | 20,046 | 1427 |
| **17** | **+** | **+** | 33,750,282 | 53,470,696 | 11,406,029 | 8,056,144 | 23,042 | 1448 |
| **18** | **-** | **-** | 29,124,395 | 48,445,743 | 10,450,504 | 7,079,538 | 16,857 | 1326 |
| **19** | **-** | **+** | 28,912,773 | 44,370,873 | 9,347,127 | 7,126,266 | 17,655 | 1323 |
| **20** | **+** | **+** | 28,631,752 | 43,695,264 | 9,408,869 | 7,035,099 | 19,082 | 1361 |
| | | | | | | | | |
| | | Average | **29,357,082** | **46,619,022** | **9,847,031** | **7,195,744** | **18,417** | |

**Figure S1 -** Non-metric multidimensional scaling (NMDS) ordinations of relationships among experimental plots based on Bray-Curtis dissimilarities calculated from: (A) relative abundance of bacterial genera, and (B) relative abundance of functional roles (the most specific level of the SEED hierarchy (Overbeek *et al.*, 2005)) of microbial protein-coding genes. This figure presents the same results as Figure 1 B and D, but at a more specific end of the taxonomy and functional hierarchies, to demonstrate that the patterns are consistent.

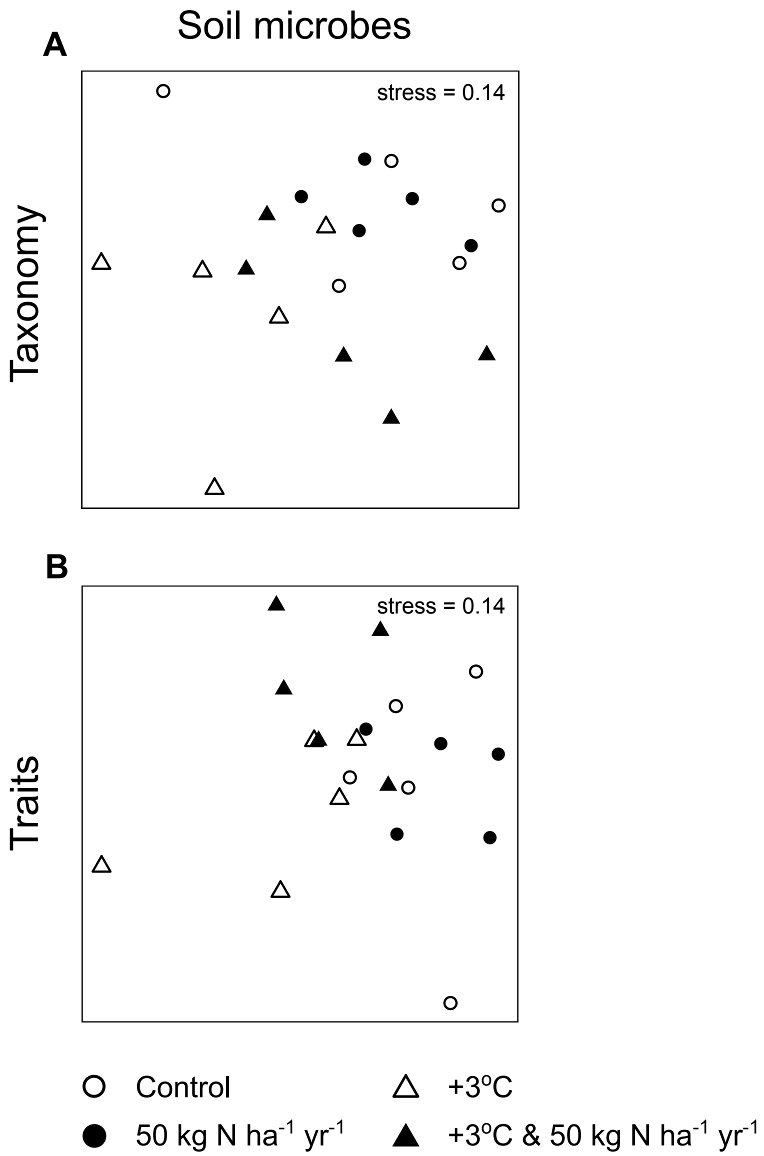

**Figure S2** – Mean relative abundance of bacterial phyla detected in five replicate plots per treatment combination. Error bars represent one standard error. Note the different scales on the y-axes.

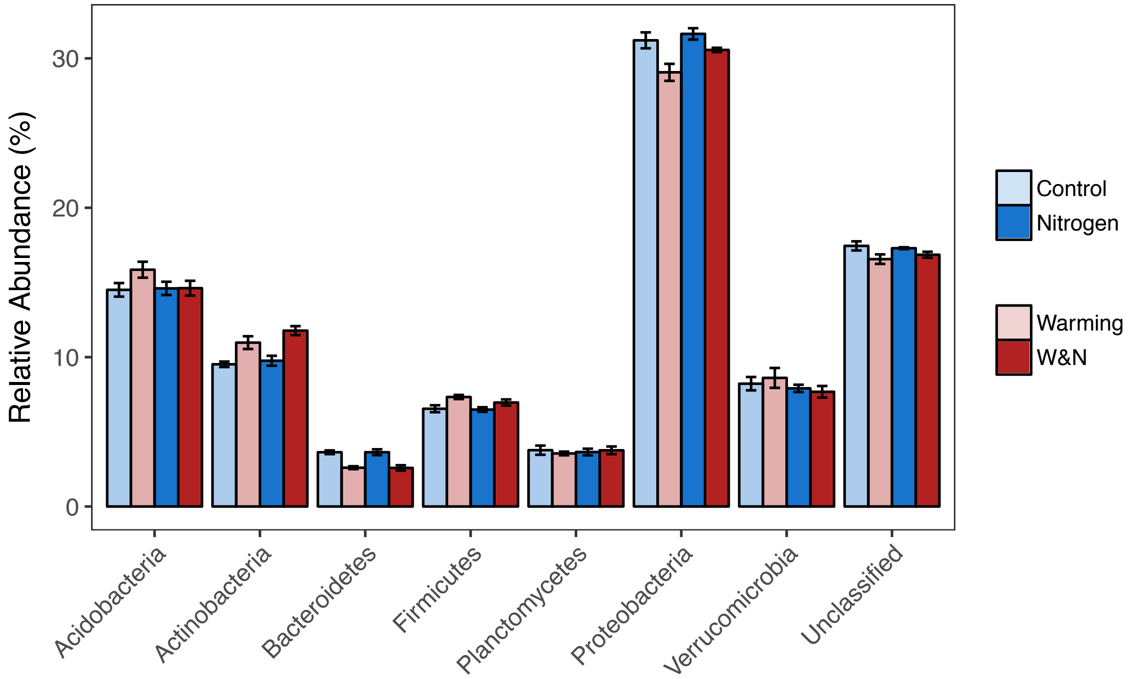


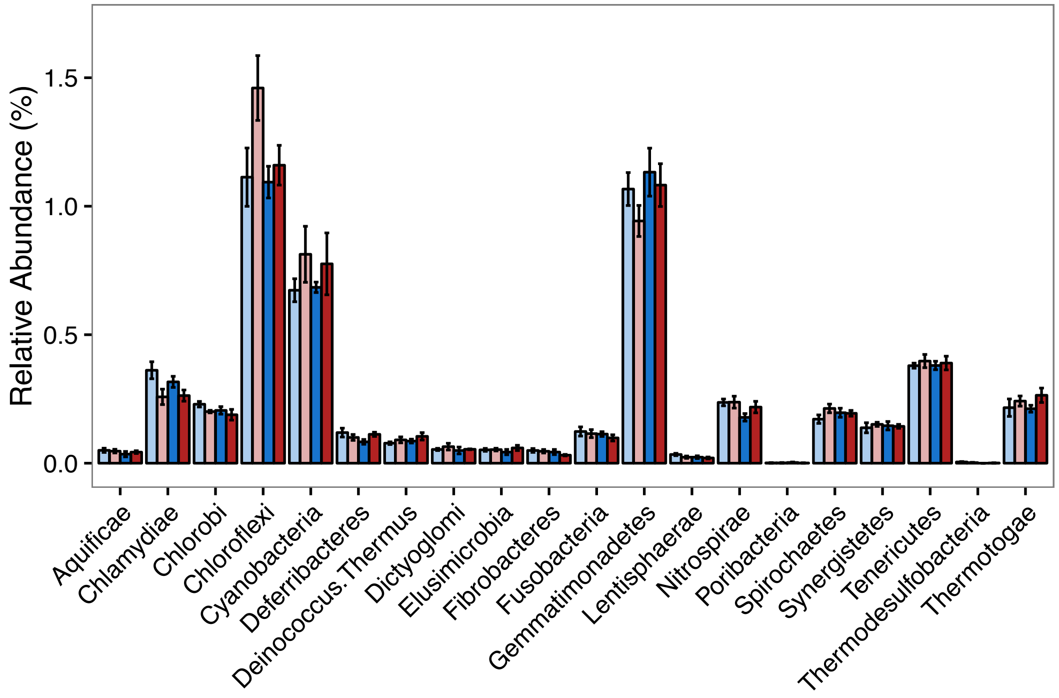

**Figure S3** – $Log_2$ of response ratios for relative abundance of bacterial phyla in experimental plots to mean relative abundance in control plots. These results are presented to assist with interpretation of the multivariate analyses in Figure 1B and Table S5. Asterisks indicate phyla significantly impacted by soil warming (red) and nitrogen addition (green) (* $p$ <0.05, ** p < 0.01 ***, $p$ < 0.001,). Six of the 26 phyla were significantly impacted by soil warming at $p$ < 0.05, and one was impacted by N addition. No significant warming by N addition interactions were observed. Despite the inflated type I error rate, the probability of observing six and one significant results of 26 tests is 0.001 and 0.376, respectively (Moran, 2003).

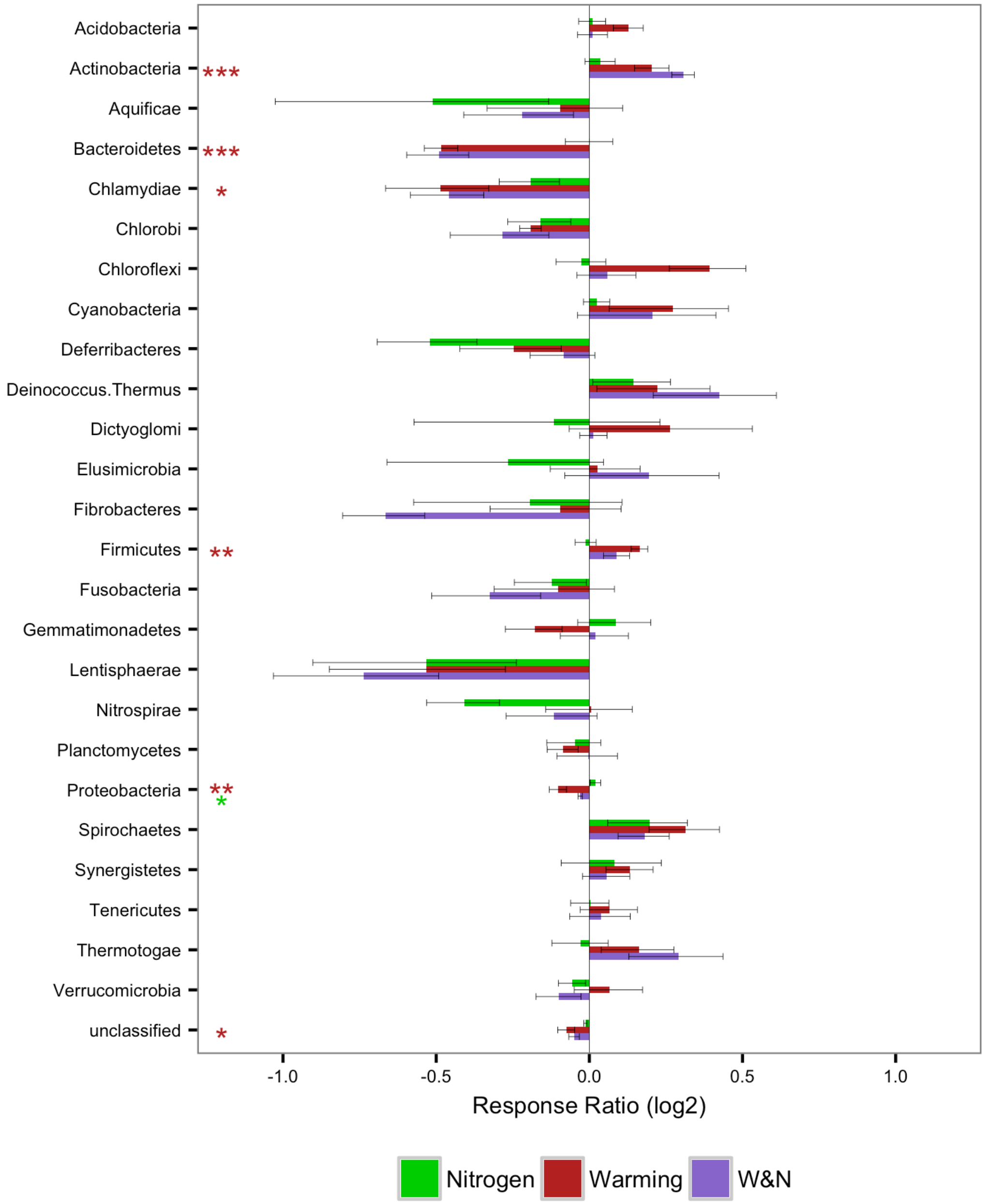

**Figure S4 -** $Log_2$ of response ratios for relative abundance of the broadest level of subsystems of bacterial protein-coding genes in experimental plots to mean relative abundance in control plots. These results are presented to assist with interpretation of the multivariate analyses in Figure 1D and Table S5. Red asterisks indicate subsystems significantly impacted by soil warming (* $p$ <0.05, ** p< 0.01). Six of the 28 subsystems were significantly impacted by soil warming at $p$ < 0.05. No significant warming by N addition interactions were observed. Despite the inflated type I error rate, the probability of observing six significant results of 28 tests is 0.002 (Moran, 2003).

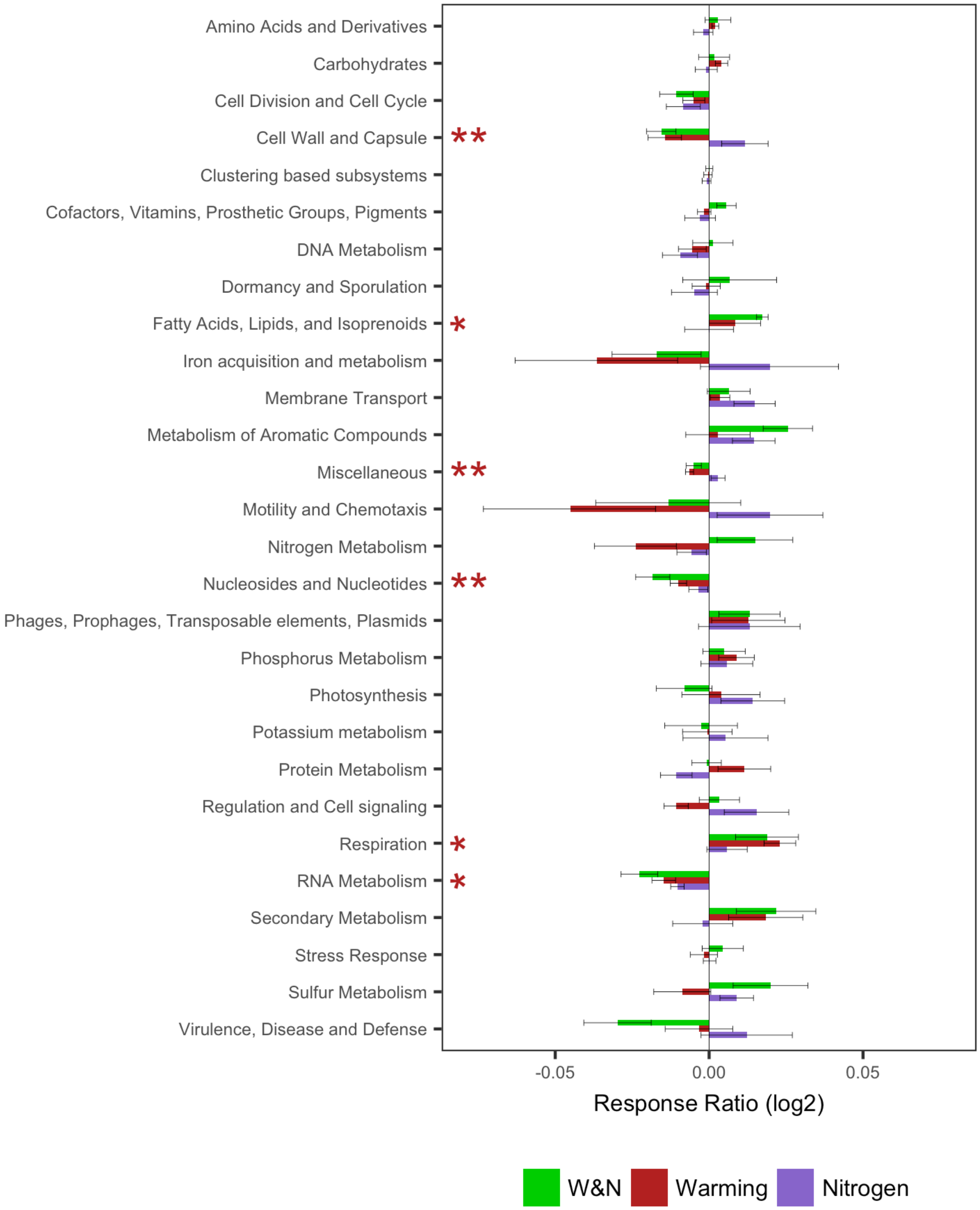

**Figure S5** - Response ratios of 'Carbohydrate' subsystems. Each treatment plot is compared to the mean of the control plots. Values equal to one indicate no difference from the control mean.

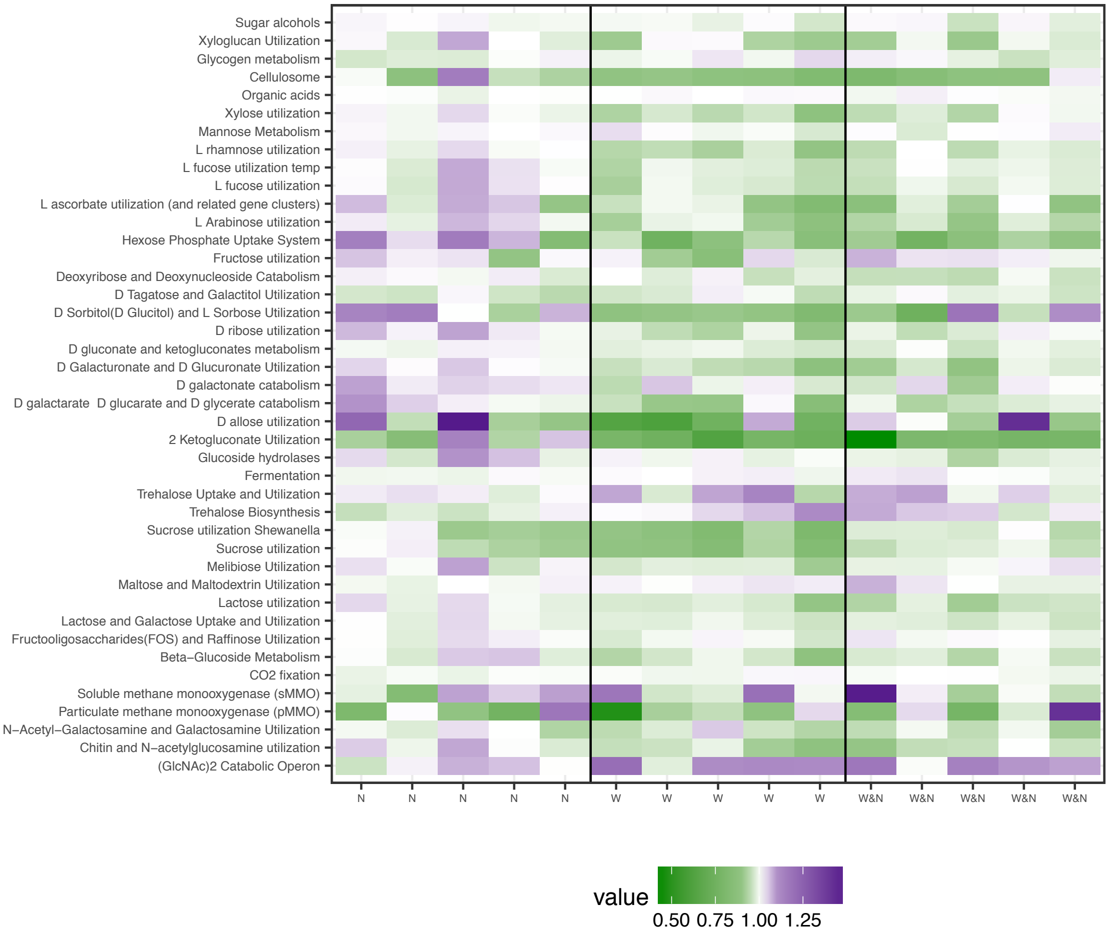

**Figure S6 -** (A) Non-metric multidimensional scaling ordination among experimental plots based on Bray-Curtis dissimilarities calculated from relative abundance of 'Nitrogen Metabolism' level three subsystems. Results of permanova analyses for these data are as follows; W (warming) : $R^2$ = 0.08, $p$ = 0.17; N (nitrogen addition): $R^2$ = 0.10, $p$ = 0.09; WxN: $R^2$ = 0.11, $p$ = 0.08. (B) Response ratios ($log_2$) of 'Nitrogen Metabolism' level three subsystems. Bars represent the mean response ratio of treatment plots compared to control plots with one standard error. Significant treatment effects from linear models are indicated with N for a significant impact of nitrogen addition, W for a significant impact of soil warming, and WxN indicates a significant interactive effect. * $p < 0.05$, ** $p < 0.01$, and *** $p < 0.001$.

**A**

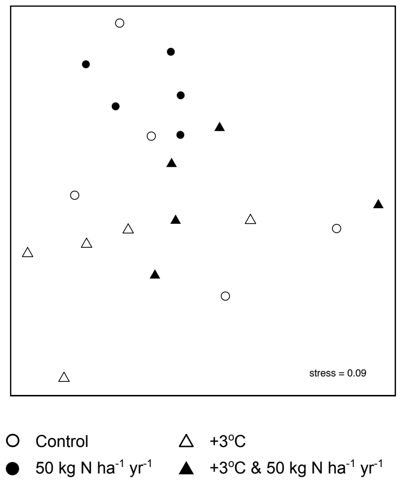


**B**

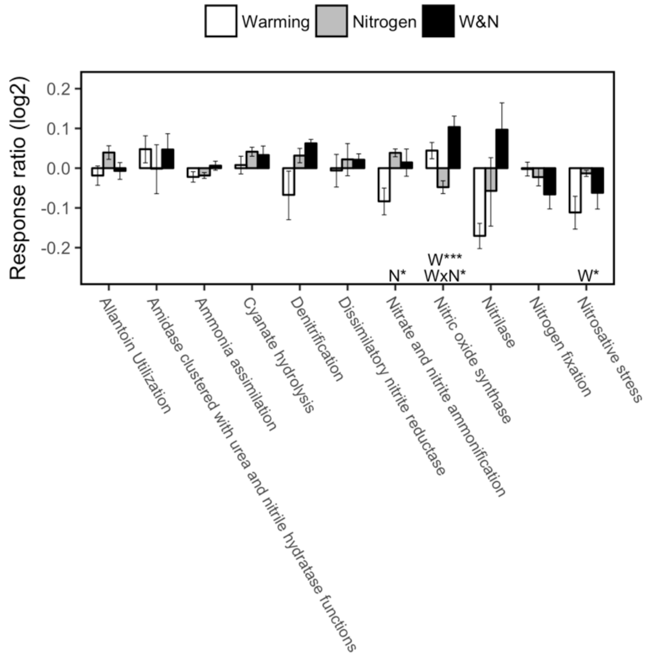

**Figure S7 -** Strength of relationships between plant and soil microbial communities. Procrustes rotations by treatment combinations. Relationships tested were between matrices of (A) cover of colonizing plants and relative abundance of bacterial phyla, and (B) standardized community weighted mean trait values of the colonizing plant species and relative abundance of the most specific level of subsystems of bacterial protein-coding genes. Correlation coefficients are listed on each plot with *p*-values in parentheses. Solid and dotted cross-hairs indicate the original and rotated axes, while the original and rotated position of each point are indicated by head and talks of arrows. Shorter arrows indicate similar distances among points between the two matrices.

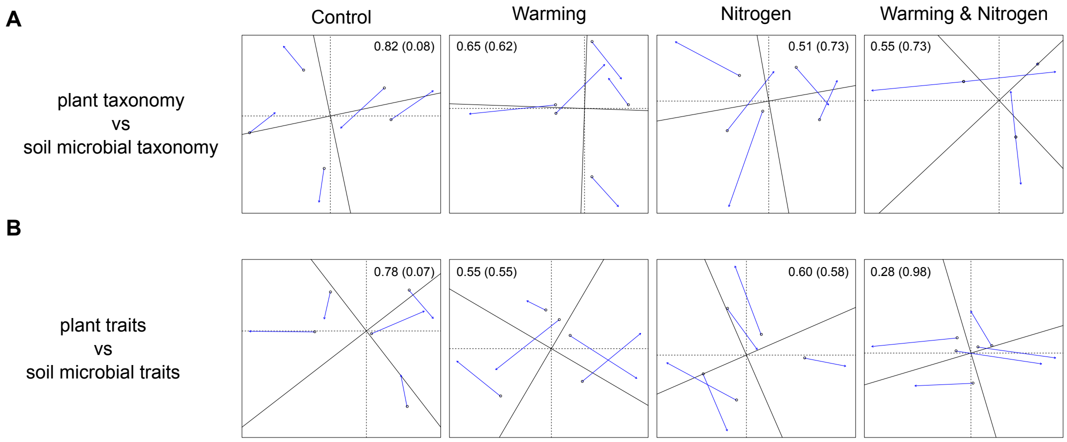

**Figure S8 -** Sequence coverage curves for soil metagenomes. The number of reads is plotted against the number of bacterial OTUs (A) and functional roles (B).

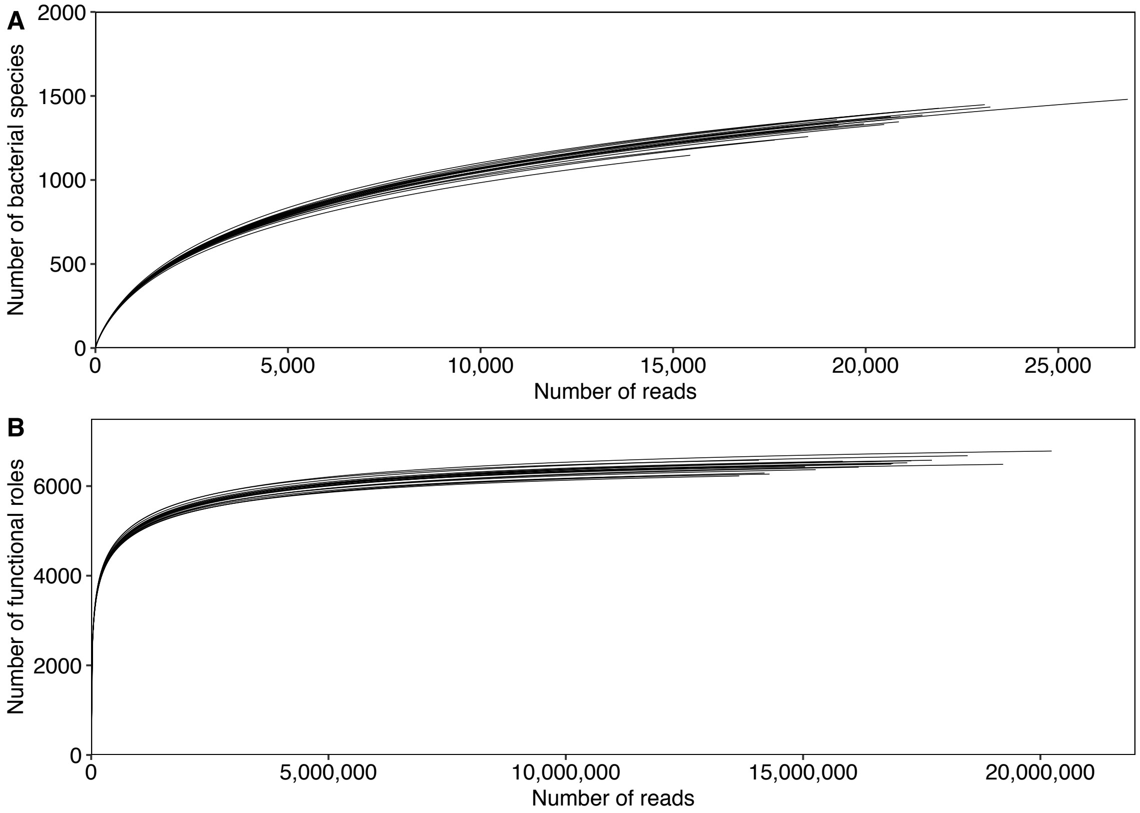

**Figure S9 -** Non-metric multidimensional scaling ordination among experimental plots based on Bray-Curtis dissimilarities calculated from relative abundance of archaeal phyla. Results of permanova analyses for these data are as follows; W (warming): $R^2$ = 0.02, $p$ = 0.79; N (nitrogen addition): $R^2$ = 0.03, $p$ = 0.59; WxN: $R^2$ = 0.01, $p$ = 0.82.

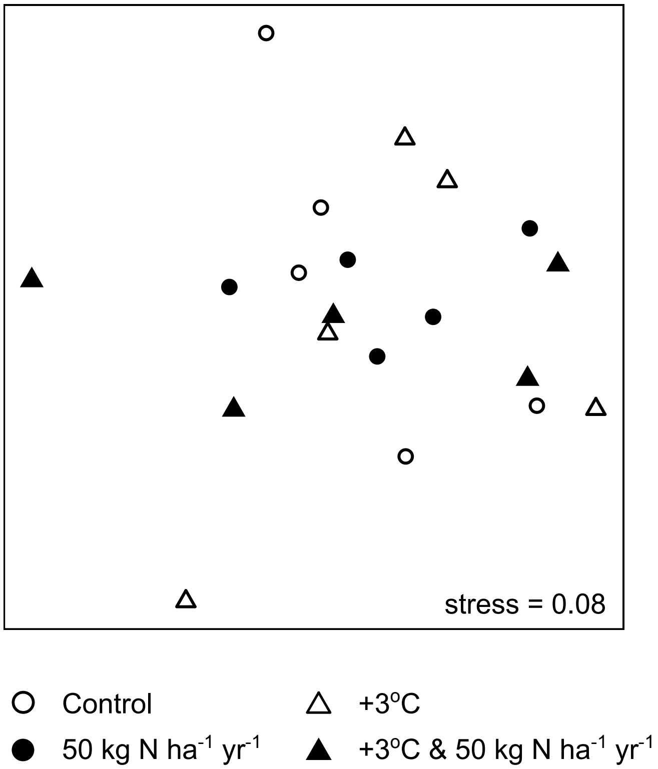

Supplement: Supplementary file 1 — Supplementary information for Above and belowground community strategies respond to different global change drivers [file 41598_2019_39033_MOESM1_ESM.pdf]
